# Effect of trace element mixtures on the outcome of patients with esophageal squamous cell carcinoma: a prospective cohort study in Fujian, China

**DOI:** 10.1186/s12885-023-11763-9

**Published:** 2024-01-02

**Authors:** Kaili Yu, Shuang Liu, Zheng Lin, Jianyu Song, Qiaoyan Zeng, Jinsong Zhou, Juwei Zhang, Suhong Zhang, Jianbo Lin, Zhisheng Xiang, Zhijian Hu

**Affiliations:** 1 Department of Epidemiology and Health Statistics, School of Public Health, Fujian Medical University, Fuzhou, 350122 Fujian China; 2 Sun Yat-Sen University Cancer Center/Cancer Hospital, Guangzhou, 510060 China; 3 The First Affiliated Hospital of Fujian Medical University, Fuzhou, 350005 Fujian China; 4 Fujian Cancer Hospital, Fuzhou, 350014 Fujian China; 5 Key Laboratory of Ministry of Education for Gastrointestinal Cancer, Fujian Medical University, FuZhou, 350122 Fujian China

**Keywords:** Trace elements, Outcome, Esophageal squamous cell carcinoma, Bayesian kernel machine regression

## Abstract

**Background:**

The evidence about the effects of trace elements on overall survival(OS) of patients with esophageal squamous cell carcinoma(ESCC) is limited. This study aims to evaluate mixed effects of plasma trace elements on OS of ESCC.

**Methods:**

This prospective cohort analysis included 497 ESCC patients with a median follow-up of 52.3 months. The concentrations of 17 trace elements were measured. We fitted Cox’s proportional hazards regression, factor analysis and Bayesian kernel machine regression (BKMR) models to estimate the association between trace elements and OS.

**Results:**

Our analysis found that in the single-element model, Co, Ni, and Cd were associated with an increased risk of death, while Ga, Rb, and Ba were associated with a decreased risk. Cd had the strongest risk effect among all elements. As many elements were found to be mutually correlated, we conducted a factor analysis to identify common factors and investigate their associations with survival time. The factor analysis indicated that the factor with high factor loadings in Ga, Ba and B was linked to a decreased risk of death, while the factor with high factor loadings in Co, Ti, Cd and Pb was associated with a borderline significantly increased risk. Using BKMR analysis to disentangle the interaction between elements in significant factors, we discovered that Ga interacted with Ba and both elements had U-shaped effects with OS. Cd, on the other hand, had no interaction with other elements and independently increased the risk of death.

**Conclusions:**

Our analysis revealed that Ga, Ba and Cd were associated with ESCC outcome, with Ga and Ba demonstrating an interaction. These findings provide new insights into the impact of trace elements on the survival of patients with ESCC.

**Supplementary Information:**

The online version contains supplementary material available at 10.1186/s12885-023-11763-9.

## Background

Esophageal cancer (EC) is the seventh most frequently diagnosed cancer and the sixth leading cause of cancer death worldwide, with heavy burden of 604,100 (3.1% of all sites) new cases and 544,076 (5.5% of all sites) new deaths annually based on global cancer 2020 [1]. Despite recent advancements in treatments for EC, the prognosis remains poor with an overall 5-year survival rate of only 7–40% [2, 3]. Esophageal squamous cell carcinoma (ESCC), which is one of the two most common histological subtypes of EC, accounts for over 90% of all EC cases in China [4]. Thus, examining the prognostic factors associated with ESCC has significant public and clinical implications for improving its prognosis.

Trace elements, which are essential components of human tissue, play a critical role in the body's metabolism and the maintenance of physiological functions. It has been established that they are essential substances for human health [5]. Accumulating epidemiological studies including several large prospective studies and comprehensive meta-analysis have shown associations between trace elements and cancer risk. For instance, the European Prospective Investigation into Cancer and Nutrition (EPIC) study, which enrolled over 521,000 study participants from10 western European countries, found that a low Se status increases the risk to develop cancer, such as colorectal cancer [6]. A comprehensive meta-analysis based on 36 studies with 5,747 female subjects showed that serum copper levels were significantly higher in breast cancer patients compared to healthy controls and those with benign breast diseases [7]. Another meta-analysis including 2,894 cases and 9,419 controls revealed that lower serum Zn may be a risk of lung cancer [8]. A few mechanism researches stated that trace elements disturbances may be involved in tumor initiation by increasing cell damage, DNA injuries, and cellular redox imbalance [9–11].

Several studies also addressed the question whether trace elements concentrations could predict the survival probability of cancer patients. Total serum selenium was proved to be correlated inversely with breast cancer mortality by a multicentre cohort study [12]. High blood levels of Cd have been identified as a significant prognostic risk factor for lung cancer [13], breast cancer [14] and nasopharyngeal carcinoma [15]. However, to date, the effect of exposure to trace elements on the overall survival (OS) of patients with ESCC has not been elucidated.

In this study, we aimed to investigate the effect of 17 trace elements on the OS of patients with ESCC. As humans are exposed to multiple elements simultaneously, we explored the single and joint effects of multiple elements on OS and the potential interactions between elements.

## Methods

### Study design and participants

Patients were recruited from October 2014 to August 2020 in the First Affiliated Hospital of Fujian Medical University and Fujian Cancer Hospital. The inclusion criteria for patients were: (1) newly diagnosed with primary ESCC, (2) aged above 18, and (3) having lived in Fujian Province for the past 10 years. The exclusion criteria were: (1) patients with other malignant tumors, (2) patients with blood disorders include anemia, bleeding disorders such as hemophilia, blood clots, and blood cancers (e.g. leukemia, lymphoma, and myeloma), (3) patients with endocrine and metabolic diseases, and (4) those diagnosed as mentally unstable.

This study was approved by the Ethics Committee of Fujian Medical University (approval number: 201495). The informed consent was provided to each subject before enrolled.

### Date collection and follow-up

Medical record reviews were conducted to collect data on demographic characteristics and clinical data. Demographic characteristics included age and gender. Clinical data included tumor location, clinical/pathological stage, treatments, T classification, N classification and M classification. The clinical/pathological stage was determined with the 8th edition American Joint Committee on Cancer tumor node metastasis (TNM) staging criteria.

A total of 531 patients with ESCC were recruited. Participants were excluded from the analysis if they (1) were diagnosed with other tumors (*n* = 21); (2) had blood disorders or endocrine and metabolic diseases (*n* = 13); (3) did not complete clinical data (*n* = 5). The final sample size was *N* = 493, which was used in the current study.

In this study, the patients with ESCC were followed up by phone call after their discharge from the hospital at a frequency of once every three months in the first year and once every six months in the second and subsequent years. The outcome measure, OS, was defined as the time from diagnosis of ESCC to death of patient or the end of follow-up. The follow-up time in this study was until September 1, 2021, with a median follow-up time of 52.3 months (ranging from 12.0 to 84.2 months). The missing rate for follow-up data was 14.6%. Based on the calculation of PASS software, the sample size could met 80% statistical power for our conclusions.

### Measurement of plasma trace elements

Fasting blood samples were collected from each patient upon admission to the hospital and prior to treatment initiation. The samples were collected after fasting overnight for at least 8 h and were temporarily stored at 4 °C before being divided into different components and stored at -80 °C in our laboratory until analysis.

We investigated 17 trace elements, namely, titanium (Ti), vanadium (V), chromium (Cr), manganese (Mn), cobalt (Co), nickel (Ni), copper (Cu), strontium (Sr), cadmium (Cd), thallium (Tl), lead (Pb), boron (B), aluminum (Al), gallium (Ga), rubidium (Rb), Cesium (Cs), and Barium (Ba) in plasma. The plasma was thawed at 4 °C, and then 0.2 mL of plasma was mixed with 3.8 mL of a diluent consisting of 0.01% (v/v) Triton and 0.1% (v/v) nitric acid. The samples were sufficiently shaken before analysis. The concentrations of the trace elements were measured together by Agilent 7700x  Inductively Coupled Plasma Mass Spectrometry (ICP-MS).

A blank sample was included with every 20 samples during analysis. The blank samples consisted of the same diluent of 0.01% (v/v) Triton and 0.1% (v/v) nitric acid used in the dilution of the plasma samples. The accuracy of the measurement was evaluated using a recovery test, with a reference range of spike-and-recovery of 80% to 120%. The limits of detection (LODs) were calculated as 3 times the standard deviation of 10 consecutive measurements of the blank diluent. In this study, the detection rates of all elements were greater than 70% based on the LODs, and the R^2^ value for the response function was exceeding 0.99 for all trace elements. The detailed data are described in Table S1 and Table S2.

### Statistical analysis

After the measurement, all 17 trace elements retained for subsequent analysis. Values of concentrations which were lower than the LODs would be replaced with LODs to fill up the vacancy in data preprocessing. Descriptive analyses were performed to describe the demographic characteristics and clinical variables of all participants. Mean, geometric mean and percentiles were calculated to describe the distribution of elements. Spearman’s correlation analysis was performed to assess bivariate relationships between trace element levels.

To approximate a normal distribution, logarithmic transformation was adopted for 17 trace elements in the following models.

### Cox’s proportional hazards regression model

We first used Cox’s proportional hazards regression models to evaluate the hazard ratios (HRs) and 95% confidential intervals (95% CIs) of each single element on OS, adjusting for potential covariates included gender, age, tumor location, clinical/pathological stage and surgery. Each element level was categorized into tertiles, with the lowest tertile was designated as reference.

### Factor analysis

Given the results of the correlations between elements, we adopted factor analysis to extract common factors from 17 elements and then explored the effects of these common factors on OS. We fit a Cox’s proportional hazards regression model for each factor, adjusting for potential covariates included gender, age, tumor location, clinical/pathological stage and surgery.

### Bayesian Kernel Machine Regression (BKMR) model

Then, we applied BKMR model to further discuss the interactions between multiple elements within the factors with OS, and visualized the bivariate exposure for every two elements.

The BKMR model is a nonparametric Bayesian variable selection framework, which can estimate the overall effects of mixture components and evaluate potential interaction effects and nonlinear associations among multiple exposures. The function of the BKMR model was as follows.

$${Y}_{i}=h\left(A,B,C,D,E,F,G\dots \right)+{\beta }^{T}{Z}_{i}+{e}_{i}$$

where ${Y}_{i}$ is a health endpoint. The function h() models the exposure–response mechanism using a Gaussian kernel, allowing for inclusion of interaction terms. A,B,C and so on represented vectors of predictor variables(trace elements). The coefficients ${\beta }^{T}$ represent the estimated effects of covariates, where ${Z}_{i}$ contains a set of covariates that need to be adjusted for(gender, age, tumor location, clinical/pathological stage and surgery). ${e}_{i}$ referred to residual.

BKMR models in this study were conducted with 10,000 iterations by Markov Chain Monte Carlo algorithm.

All data were analyzed using SPSS software (version 25.0. IBM Corp., Armonk, NY, USA.) and R software (version 3.6.3, R Foundation for Statistical Computing.). Overall, the two-sided *p* value < 0.05 was considered as statistically significant.

## Results

### Demographic and clinical characteristics of patients with ESCC

The characteristics of the study population are described in Table 1. The mean age at ESCC diagnosis was 61.5 years. About three-quarters of participants were male and one-quarter were female. The most common location of the tumor was the middle thoracic region. Approximately 59.0% (*n* = 291) of patients were diagnosed with stage III-IV at the time of diagnosis, and around 80% of them received surgical treatment.

**Table 1:** Characteristics of the study population (*N* = 493)

Characteristics	N (%)
Age,year	61.46 ± 8.5
< 60	206 (41.8)
≥ 60	287 (58.2)
Gender	
Female	122 (24.7)
Male	371 (75.3)
Tumor location	
Cervical esophagus-upper thoracic	86 (17.4)
Middle thoracic	240 (48.7)
Lower thoracic	167 (33.9)
Clinical/Pathological Stage	
I-II	202 (41.0)
III-IV	291 (59.0)
Surgery	
Yes	386 (78.3)
No	107 (21.7)
Grade^a^	
Well	39 (7.9)
Moderately	257 (52.1)
Poorly/undifferentiated	90 (18.3)
T classification	
T1-T2	176 (35.8)
T3-T4	293 (59.3)
N classification	
N0	243 (49.3)
N1-N2	147 (29.9)
N3	79 (15.9)
M classification	
M0	469 (95.1)
M1	24 (4.9)

*ESCC* esophageal squamous cell carcinoma

^a^Only surgical patients had information about grade

### Plasma trace element concentrations in patients with ESCC

**Fig. 1:**
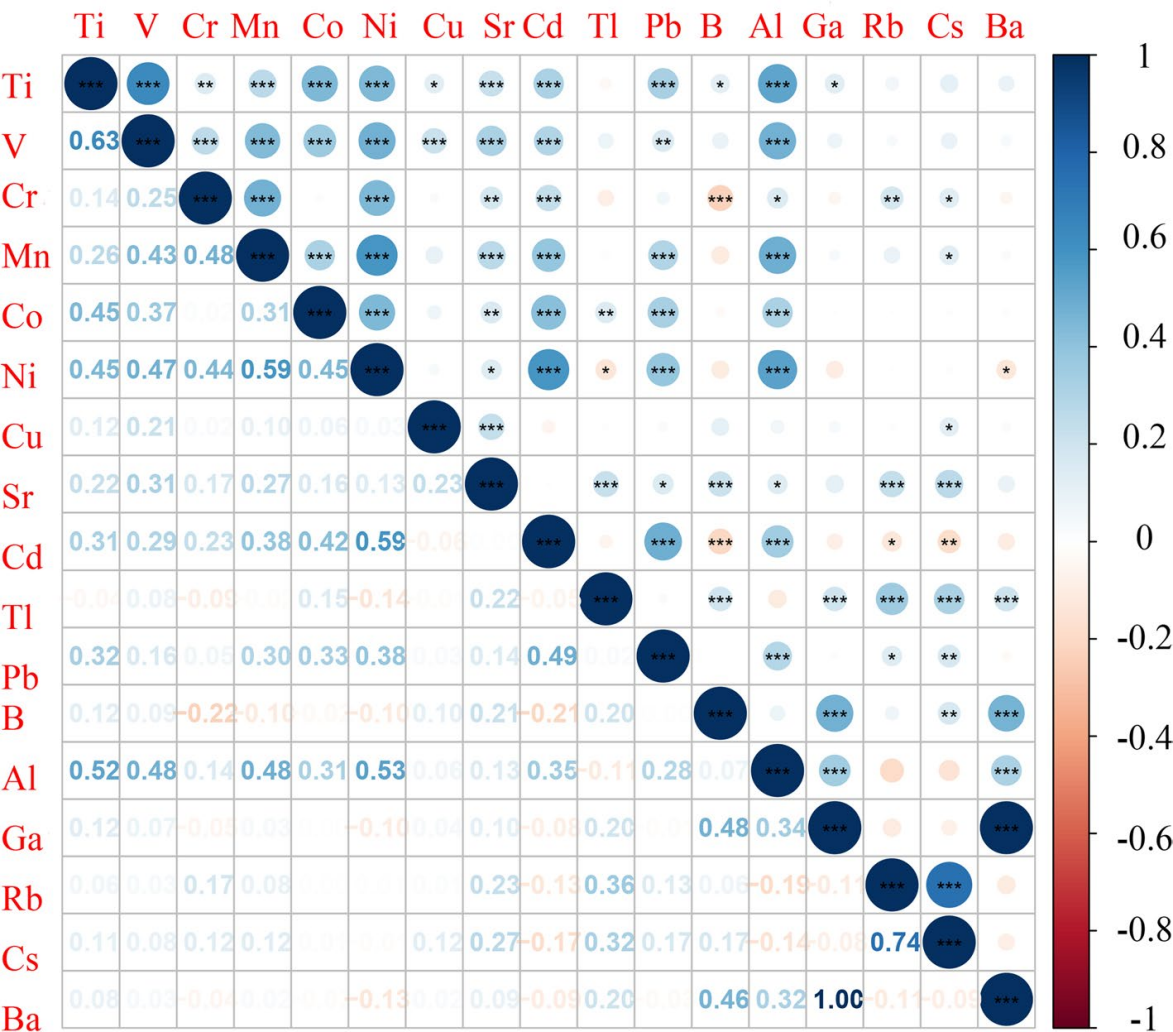
The Spearman correlation heatmap of trace elements’ levels among the participants. * *P* < 0.05; ** *P* < 0.01; *** *P* < 0.001

The distributions of the 17 plasma trace elements were summarized in Table 2 and the detection rates of all elements were higher than 70%. Spearman correlations among the 17 trace elements were presented in Fig. 1. It is clear that most elements had mutual correlations with other elements (*P* < 0.05), and the absolute value of the coefficients ranged from 0.12 to 1.00. The strongest correlation was observed between Ga and Ba (*R* = 1.00, *P* < 0.001).

**Table 2:** Distribution of 17 elements in patients with ESCC (*N* = 493,unit: ug/L)

Element^a^	Mean	Geometric mean	Percentile				
			2.5	25	50	75	97.5
47Ti	35.94	34.27	18.43	28.02	33.59	41.27	64.63
51 V	1.12	0.27	0.01	0.01	0.81	1.48	4.98
53Cr	2.29	1.53	0.02	1.22	1.73	2.52	5.61
55Mn	2.84	1.40	0.01	1.02	2.00	3.43	9.45
59Co	0.24	0.22	0.09	0.17	0.22	0.27	0.58
60Ni	2.76	2.22	0.47	1.42	2.38	3.63	7.12
63Cu	964.52	924.70	545.41	774.91	897.68	1108.49	1697.61
88Sr	47.94	45.17	23.43	36.31	45.15	57.30	88.81
111Cd	0.75	0.14	< 0.01	0.06	0.15	0.54	6.52
205Tl	0.15	0.05	< 0.01	0.04	0.09	0.18	0.72
208Pb	14.77	8.40	1.24	5.16	8.56	15.86	61.44
11B	47.72	22.20	1.00	14.90	26.63	43.65	362.57
27Al	117.26	61.42	0.40	37.93	77.41	146.03	503.98
69 Ga	8.54	6.49	0.91	3.80	7.28	12.12	22.86
85Rb	820.23	687.82	273.55	478.70	644.66	908.11	2840.36
133Cs	1.80	1.58	0.63	1.08	1.62	2.16	4.34
137Ba	38.61	29.47	3.98	17.77	32.65	54.00	100.28

*ESCC* esophageal squamous cell carcinoma

^a^The number before the element represents the isotope of each element

### Association of plasma elements with overall survival in ESCC using single-element models

We firstly estimated the associations between each element level and OS of ESCC using Cox’s proportional hazards regression model. The concentration of each element (transformed by natural logarithm) was grouped according to their tertiles, and the lowest concentration group (tertile 1) was taken as the reference in the model. After controlling for potential confounders (age,gender, tumor location, clinical/pathological stage, surgery), we found that the highest tertile of Co, Ni, and Cd were associated with an increased risk of death (tertile3 vs. tertile1:HR = 1.591, 95% CI: 1.105 ~ 2.291; HR = 1.404, 95% CI: 0.977 ~ 2.017; HR = 1.851, 95% CI: 1.258 ~ 2.723). Cd is obviously the element with the strongest risk effect among all elements. Conversely, Ga, Rb and Ba were associated with a decreased risk of death (all *P* trend < 0.05). Statistical significance was found in each tertile for Ba (tertile2 vs. tertile1: HR = 0.679, 95% CI: 0.480 ~ 0.961;tertile3 vs. tertile1: HR = 0.659, 95% CI: 0.455 ~ 0.956). Negative associations were only observed in the tertile2 for Rb exposure (tertile2 vs. tertile1: HR = 0.591, 95% CI: 0.408 ~ 0.857) and in the tertile3 for Ga exposure (tertile3 vs. tertile1:HR = 0.640, 95% CI: 0.438 ~ 0.934) (Table 3, Table S3).

**Table 3:** Cox proportional hazard analysis for the association between each element and survival time of patients with ESCC^a^

Elements (ug/L)	Tertile1	Tertile2	Tertile3	*P* trend
47Ti	1	1.104 (0.768 ~ 1.586)	0.897 (0.629 ~ 1.277)	0.534
51 V	1	1.022 (0.699 ~ 1.495)	0.993 (0.676 ~ 1.458)	0.985
53Cr	1	0.933 (0.648 ~ 1.344)	0.826 (0.572 ~ 1.191)	0.582
55Mn	1	1.490 (1.032 ~ 2.150)	1.127 (0.768 ~ 1.655)	0.076
**59Co**	**1**	**1.216 (0.835 ~ 1.771)**	**1.591 (1.105 ~ 2.291)**	**0.039**
**60Ni**	**1**	**0.903 (0.618 ~ 1.319)**	**1.404 (0.977 ~ 2.017)**	**0.035**
63Cu	1	1.082 (0.746 ~ 1.570)	1.232 (0.848 ~ 1.789)	0.533
88Sr	1	1.055 (0.728 ~ 1.527)	0.895 (0.614 ~ 1.305)	0.666
**111Cd**	**1**	**1.372 (0.939 ~ 2.004)**	**1.851 (1.258 ~ 2.723)**	**0.007**
205Tl	1	0.842 (0.586 ~ 1.209)	0.804 (0.555 ~ 1.165)	0.479
208Pb	1	1.050 (0.730 ~ 1.510)	1.447 (1.005 ~ 2.085)	0.090
11B	1	0.802 (0.563 ~ 1.141)	0.787 (0.548 ~ 1.130)	0.329
27Al	1	0.890 (0.620 ~ 1.277)	1.074 (0.753 ~ 1.532)	0.620
**69 Ga**	**1**	**0.719 (0.510 ~ 1.014)**	**0.640 (0.438 ~ 0.934)**	**0.042**
**85Rb**	**1**	**0.591 (0.408 ~ 0.857)**	**0.830 (0.584 ~ 1.181)**	**0.019**
133Cs	1	1.067 (0.732 ~ 1.556)	0.940 (0.657 ~ 1.345)	0.790
**137Ba**	**1**	**0.679 (0.480 ~ 0.961)**	**0.659 (0.455 ~ 0.956)**	**0.034**

*ESCC* esophageal squamous cell carcinoma

^a^Each element was calculated in the model separately and models was adjusted by gender, age, tumor location, clinical/pathological stage and surgery

### Multiple elements exposures model for OS of ESCC

As many elements were found to be mutually correlated, we conducted a factor analysis to identify common factors and investigate their associations with survival time. The results showed a Kaiser–Meyer–Olkin (KMO) value of 0.658 and a significant chi-square value of 3878.812 (*P* < 0.001) on Bartlett's sphericity test, indicating that the factor analysis model was appropriate for exploring and analyzing the impact factors.

The principal component analysis method was used to extract common factors, and the maximum variance method was used for orthogonal rotation. According to the principle that the eigenvalue was greater than 1, we extracted six factors that explain 70.482% of the total variance, as shown in Figure S1 and Table S4. Table 4 presented the factor loading of each factor.

**Table 4:** Rotated component matrix

Factors	Factor loading
1	Ga(0.968), Ba(0.966), B(0.619)
2	Co(0.764), Ti(0.682), Cd(0.667), Pb(0.612)
3	Rb(0.893), Cs(0.892)
4	Mn(0.754), Ni(0.670), Al(0.610)
5	Cr(0.827), V(0.663)
6	Cu(0.830), Sr(0.519)

**Fig. 2:**
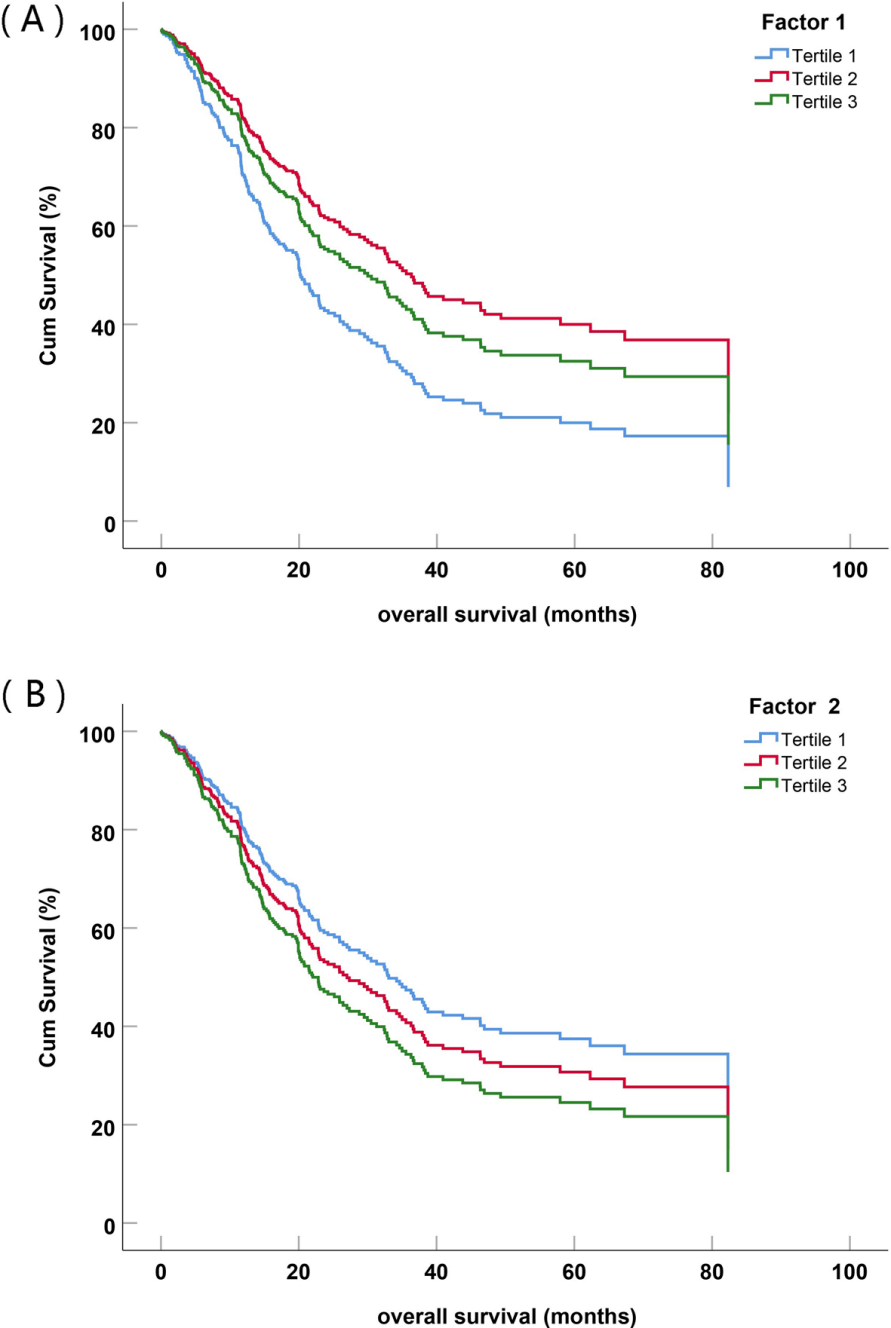
The survival function for (**A**) Factor 1 and (**B**) Factor 2

We brought six factors into the Cox model separately and adjusted for potential covariates. The scores of each factor was grouped into tertiles, with the lowest score group (tertile 1) used as the reference in the model. The results showed that factor 1 was associated with a decreased risk of death, with the strongest protective effect seen in tertile 2 followed by tertile 3. (tertile 2 vs. tertile 1:HR = 0.552, 95%CI: 0.383–0.794, *P* = 0.001; tertile 3 vs. tertile 1: HR = 0.624, 95%CI: 0.428–0.909, *P* = 0.014). Comparably, factor 2 was associated with a borderline significant increase in the risk of death for patients, with a trend suggesting that higher tertiles of factor 2 were associated with stronger harmful effects (tertile 3 vs. tertile 1: HR = 1.447, 95%CI: 0.987–2.123, *P* = 0.058) (Table 5, Table S5 and Fig. 2).

**Table 5:** Cox proportional hazard analysis for the association between extracting common factors and survival time of patients with ESCC

Characteristics	HR(95%CI)	P
Factor1		
Tertile 1	1	
Tertile 2	0.552 (0.383 ~ 0.794)	0.001
Tertile 3	0.624 (0.428 ~ 0.909)	0.014
Factor2		
Tertile 1	1	
Tertile 2	1.324 (0.916 ~ 1.914)	0.136
Tertile 3	1.447 (0.987 ~ 2.123)	0.058
Factor3		
Tertile 1	1	
Tertile 2	0.957 (0.652 ~ 1.406)	0.824
Tertile 3	0.998 (0.682 ~ 1.46)	0.990
Factor4		
Tertile 1	1	
Tertile 2	0.896 (0.618 ~ 1.298)	0.561
Tertile 3	1.063 (0.725 ~ 1.558)	0.755
Factor5		
Tertile 1	1	
Tertile 2	0.674 (0.446 ~ 1.017)	0.060
Tertile 3	0.971 (0.663 ~ 1.423)	0.882
Factor6		
Tertile 1	1	
Tertile 2	0.836 (0.576 ~ 1.213)	0.345
Tertile 3	1.014 (0.687 ~ 1.497)	0.943

As shown in Table 4, Factor 1 had a high factor loading for Ga, Ba, and B, while Factor 2 had a high factor loading for Co, Ti, Cd, and Pb. The results for Ga, Ba, Co, and Cd were consistent with the results obtained from the single-element models.

### The mutual effect between every two elements on OS

We further applied the BKMR model to explore the mutual effects and nonlinear effects between each pair of elements in factor 1 and factor 2 on OS. BKMR models were fitted to assess the effects on survival at 1, 3, and 5 years. The posterior inclusion probability (PIP) value of each element in each model is shown in Table S6 and S7. A PIP threshold of 0.5 is usually used to determine whether it is important [16]. The results showed that Ga, Ba, and Cd were the most important elements.

**Fig. 3:**
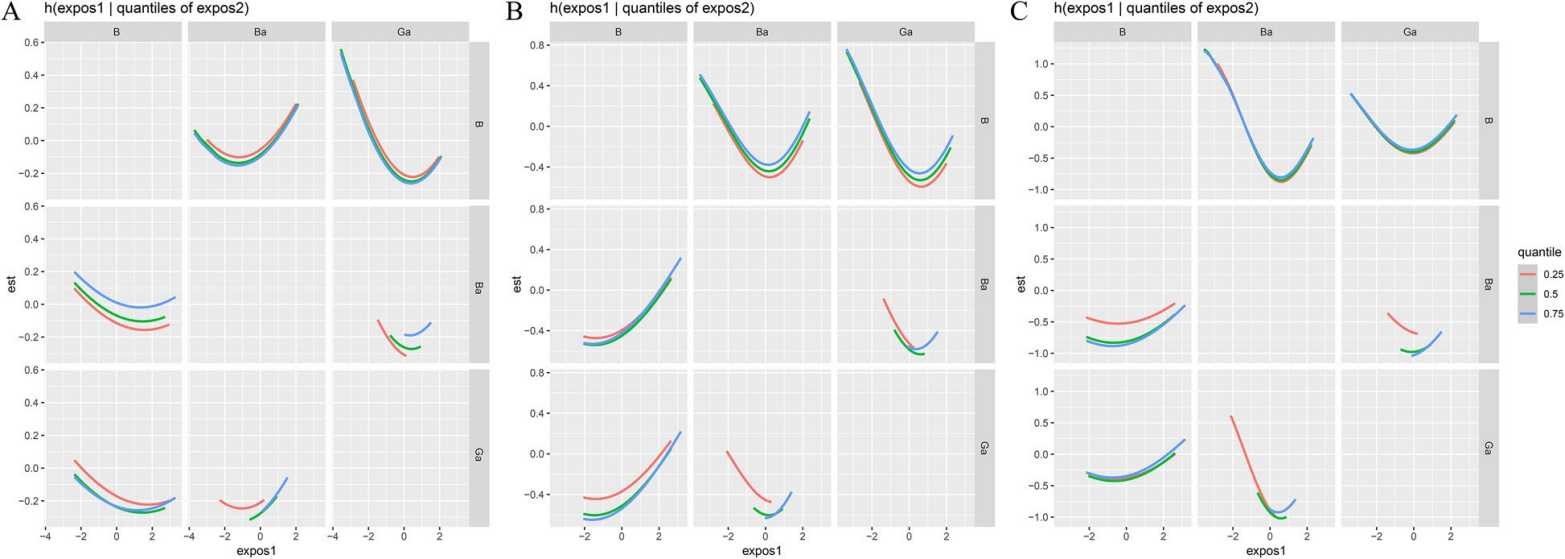
In factor1, the bivariate cross-section effects of the exposure–response function of a single element where the second element was fixed at P25, P50 and P75. **A** Outcome: survival at 1 year; **B** Outcome: survival at 3 year; **C** Outcome: survival at 5 year

**Fig. 4:**
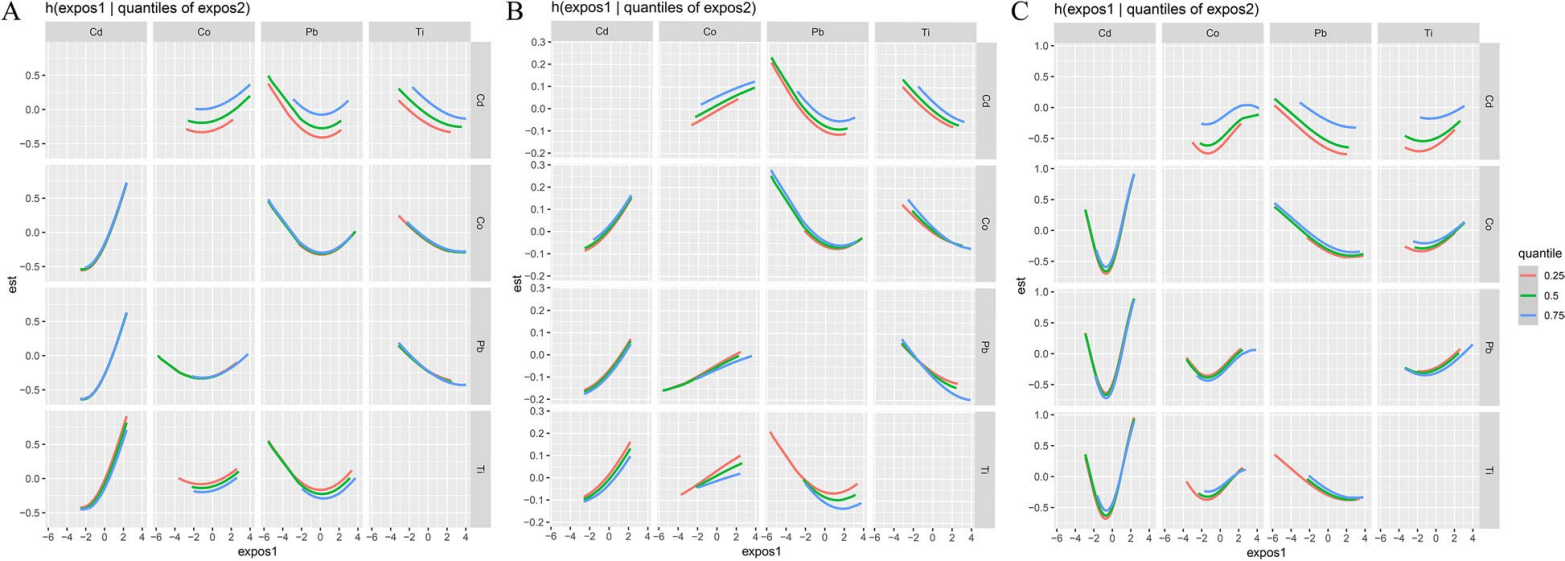
In factor2, the bivariate cross-section effects of the exposure–response function of a single element where the second element was fixed at P25, P50 and P75. **A** Outcome: survival at 1 year; **B** Outcome: survival at 3 year; **C** Outcome: survival at 5 year

We explored bivariate exposure–response consequences. Just like it shown in Figs. 3 and 4, each column was the studied element (“exposure 1”), each row represented “exposure 2”, and others were fixed at their medians. “Exposure 2” were located at their P25, P50 and P75. In Fig. 3, we found an interaction between Ga and Ba in their association with survival: the slope for Ga changed in case of Ba concentrations increasing from 25th to75th concentration, and the increasing concentrations of Ga could also change the slope for Ba. On the whole, the joint effect of Ga and Ba was significantly protective, which were consistent with the Cox’s proportional hazards regression results. Ga and Ba showed U-shaped effects with OS in the BKMR models. In Fig. 4, it is obviously that with the increasing percentiles of Cd exposure, the hazardous effects of all other elements increased almost parallelly. This indicates that Cd had no mutual effects with other elements and could increase the risk of death independently.

## Discussion

This study explored (1) the association between single element and OS of ESCC; (2) the mixed impact of multiple elements; and (3) the interaction between different elements. In the single-element model, Co, Ni, and Cd were associated with an increased risk of death, while Ga, Rb, and Ba were associated with a decreased risk of death. By extracting common factors from 17 elements, we found that factor with high factor loading in Ga, Ba and B was associated with a lower risk of death, while the factor with high factor loading in Co,Ti,Cd and Pb was associated with a borderline significantly increased risk of death in patients. Using the BKMR model, we clearly observed that Ga had a strong interaction with Ba, and they exhibited U-shaped effects with OS. Cd had no interaction with other elements and could independently increase the risk of death.

Cd is a toxic non-essential transition metal that exists in the natural environment, mainly due to agricultural and industrial pollution [17]. Smoking [18, 19], ingestion of contaminated food or water [20] and occupational exposure are the main sources of Cd exposure. Several epidemiological studies have shown that Cd exposure is a risk factor for cancer occurrence [21–23]. The International Agency for Research on Cancer (IARC) has classified Cd as a class 1 carcinogen. In addition, a systematic review and meta-analysis including nine cohort studies with 1332 cancer deaths concluded that Cd appears to be associated with increased cancer mortality [24]. WATANABE Y et al. [25] reported that even in areas without Cd pollution, the increase of Cd content in the body was significantly associated with the increased mortality of all malignant tumors. Apart from mortality, a high blood Cd level was well-reported to be a significant prognostic risk factor for lung cancer [13], breast cancer [14] and nasopharyngeal carcinoma [15] (HR = 1.56, 95% CI: 1. 02 ~ 2.36; HR = 2.247, 95% CI: 1.109 ~ 4.550; HR = 4.11, 95% CI: 1.92 ~ 8.81). These studies suggested that Cd is associated with the prognosis of various cancers. In line with these findings, our study found that Cd was a risk factor for shorter survival time in patients with ESCC. In our single-element model, Cd had the strongest risk effect among the 17 elements (HR = 1.851, 95% CI: 1.258 ~ 2.723). In BKMR models, Cd had no interaction with other elements and could increase the risk of death independently. From the current research on biological mechanism, it may be comprehensible and explicable: Firstly, Cd can induce oxidative stress to generate ROS, and excess ROS can promote the activation of proto-oncogenes by reducing the antioxidant capacity of cells [26]. Secondly, Cd alters the permeability inside mitochondria by generating ROS, activating apoptosis, mutating mtDNA, altering gene expression, inhibiting respiratory chain complexes, and reducing ATP synthesis, leading to the development of disease [27, 28]. Thirdly, Cd can inhibit DNA repair and reduce the activity of enzymes involved in eliminating damage [29], etc.

Ga is a Group IIIa metal and is regarded as a non-essential trace element. Our study presented that Ga in plasma has a protective effect on the prognosis of patients with ESCC. Consistent with our findings, clinical trials have shown that gallium salts have antineoplastic activity against non-Hodgkin's lymphoma and urothelial cancers [30–32]. It was reported that already in the 1970s, animal studies have showed the ability of Ga to concentrate in certain cancers in vivo and inhibit the growth of malignant tumor [33]. Years later, gallium compounds have been extensively studied as an anticancer agent in cancer therapy for its great antitumor efficacy and broad spectrum of antineoplastic activity in human [34]. The mechanisms of action of gallium were tracked as follows: on the one hand, Ga shares certain chemical properties with iron, which allow it to bind iron-containing proteins, perform iron-mimetic functions, and disrupt the growth of iron-dependent tumor cells [35]; on the other hand, gallium compounds can induce cell apoptosis via mitochondrial pathway by inducing cell overexpression of proapoptotic protein Bax [36]. Moreover, the complex of Ga can also directly inhibit the complex in the mitochondrial respiratory chain, resulting in the blocking of aerobic respiration process and causing cell apoptosis [37, 38]. Although the positive effect of Ga was consistently verified, our study additionally found that Ga showed a U-shaped effect with OS, which suggests that there is an optimal concentration for the strongest effect. The specific metabolic pathway is still unclear, and more in-depth research is needed to explore the mechanism.

A significant protective effect of Ba on OS was also found in this study. Ba is not recognized to have a biological role in humans [39] and its carcinogenic toxicity of barium is rather limited. Unlike other heavy metals, the Department of Health and Human Services and the International Agency for Research on Cancer have not assessed Ba as a carcinogenic species yet [40]. Jia-liu He reported the positive effect of Ba: Ba can reduce the risk of goiter by inhibiting the levels of T3 and T4 [41]. Our study showed that higher plasma Ba levels in patients with ESCC were associated with a better prognosis. Based on the interaction of Ga and Ba in their association with survival, we speculated that one of the reasons for the protective effect of Ba might be affected by Ga. Further studies are needed to clarify the mechanism between Ba and tumor development.

Our study had several strengths. To the best of our knowledge, our research is the first one to analyze the effects of so many elements on OS of patients with ESCC. And the large sample size is also a advantage. Second, we combined conventional statistical methods and a novel statistical method to distengle single and mixed effects of plasma trace elements on OS of patients with ESCC. Various statistical methods could be complementary and evaluate the effects from multiple perspectives. On one hand, we can know about the joint effect of several elements through their common factors; on the other hand, BKMR model allowed us to find the interactions of elements and the nonlinear exposure–effect in relation to OS. The study also had several limitations. First, we only measured the plasma elements at a single time point, which ignored the changes of the elements. Combine the measurements of various time points and evaluate the effects are needed in the future. Second, although a number of elements were explored in this study, there may still be some important elements which were associated with prognosis that have not been analyzed.

In summary, the findings of our study demostrated the effects of plasma trace elements on OS of patients with ESCC, which give evidence that can be used in future epidemiological and experimental studies. The clinical significance of our findings is also substantial, particularly for the treatment and care of patients with ESCC. These results suggest that monitoring and controlling the levels of some trace elements in ESCC patients may be crucial for their prognosis and overall well-being. Clinical interventions and strategies aimed at increasing or reducing exposure of some trace elements may have a significant impact. Further research into the underlying mechanisms is essential for the development of targeted interventions that can translate these findings into clinical benefits.

## Conclusions

This study indicates that exposure to multiple elements is associated with the OS of patients with ESCC. Based on our analysis of the independent and joint effects of multiple elements, we found that Ga and Ba were associated with a decreased risk of death, while Cd was associated with an increased risk of death among patients with ESCC. Additionally, Ga interacted with Ba, and both elements showed U-shaped effects on OS.

### Supplementary Information

**Additional file 1:** **Table S1.** Linearity and Detection Limits of Trace Elements Analysis Method. **Table S2.** Assessment of Spike Levels and Recovery Rates for Trace Elements. **Table S3.** Cox proportional hazard analysis for the association between each element and survival time of patients with ESCC. **Table S4.** Total variance explained. **Table S5.** Cox proportional hazard analysis for the association between extracting common factors and survival time of patients with ESCC. **Table S6.** PIP value of each element in factor 1. **Table S7.** PIP value of each element in factor 2.

**Additional file 2:** **Fig. S1.** Scree plot.

## Data Availability

All data generated or analysed during this study are included in this published article and its supplementary information files.

## Abbreviations

BKMR: Bayesian Kernel Machine Regression Model
EC: Esophageal cancer
ESCC: Esophageal squamous cell carcinoma
OS: Overall survival
