# Supplementary material for: Effect of trace element mixtures on the outcome of patients with esophageal squamous cell carcinoma: a prospective cohort study in Fujian, China
Source: BMC Cancer. 2024 Jan 2;24:24. doi: 10.1186/s12885-023-11763-9 (PMC10762846; doi:10.1186/s12885-023-11763-9)
Supplement: Supplementary file 1 — Additional file 1: Table S1. Linearity and Detection Limits of Trace Elements Analysis Method. Table S2. Assessment of Spike Levels and Recovery Rates for Trace Elements. Table S3. Cox proportional hazard analysis for the association between each element and survival time of patients with ESCC. Table S4. Total variance explained. Table S5. Cox proportional hazard analysis for the association between extracting common factors and survival time of patients with ESCC. Table S6. PIP value of each element in factor 1. Table S7. PIP value of each element in factor 2. [file 12885_2023_11763_MOESM1_ESM.docx]

# Supplemental Material

Table S1 Linearity and Detection Limits of Trace Elements Analysis Method

| Trace  element | R^2^ | LOD (μg/L) | > LOD (%) | Trace  element | R2 | LOD (μg/L) | > LOD  (%) |
| --- | --- | --- | --- | --- | --- | --- | --- |
| 47Ti | 1.0000 | 0.07 | 100.00 | 205Tl | 0.9901 | 0.00 | 73.21 |
| 51V | 1.0000 | 0.01 | 100.00 | 208Pb | 1.0000 | 0.01 | 100.00 |
| 53Cr | 0.9999 | 0.06 | 73.21 | 11B | 0.9965 | 0.87 | 83.12 |
| 55Mn | 1.0000 | 0.01 | 93.46 | 27Al | 0.9983 | 0.48 | 100.00 |
| 59Co | 0.9983 | 0.00 | 99.37 | 69Ga | 1.0000 | 0.02 | 100.00 |
| 60Ni | 1.0000 | 0.01 | 99.79 | 85Rb | 0.9999 | 0.02 | 100.00 |
| 63Cu | 1.0000 | 0.47 | 100.00 | 133Cs | 1.0000 | 0.02 | 99.79 |
| 88Sr | 1.0000 | 0.01 | 100.00 | 137Ba | 1.0000 | 0.04 | 100.00 |
| 111Cd | 1.0000 | 0.01 | 71.31 |  |  |  |  |

Table S2 Assessment of Spike Levels and Recovery Rates for Trace Elements

| Trace  element | Validation Range  (ug/L) | Spike level 1 | | Spike level 2 | | Spike level 3 | |
| --- | --- | --- | --- | --- | --- | --- | --- |
|  |  | value (ug) | recovery (%) | value (ug) | recovery (%) | value (ug) | recovery (%) |
| 47Ti | 0.494-5.814 | 2 | 97.775 | 10 | 100.010 | 40 | 103.247 |
| 51V | 0.028-5.141 | 2 | 102.618 | 10 | 101.204 | 40 | 105.490 |
| 53Cr | 0.002-5.363 | 2 | 102.013 | 10 | 97.764 | 40 | 102.236 |
| 55Mn | 0.002-5.353 | 2 | 91.686 | 10 | 95.852 | 40 | 102.221 |
| 59Co | <0.001-5.353 | 2 | 97.161 | 10 | 93.735 | 40 | 115.782 |
| 60Ni | 0.007-5.276 | 2 | 94.374 | 10 | 93.129 | 40 | 95.045 |
| 63Cu | 5.365-35.339 | 2 | 106.007 | 10 | 108.985 | 40 | 119.749 |
| 88Sr | 0.711-7.117 | 2 | 108.792 | 10 | 106.542 | 40 | 109.252 |
| 111Cd | <0.001-4.369 | 2 | 100.390 | 10 | 98.428 | 40 | 101.051 |
| 205Tl | <0.001-4.193 | 2 | 109.904 | 10 | 107.008 | 40 | 113.872 |
| 208Pb | 0.060-22.996 | 2 | 112.504 | 10 | 105.745 | 40 | 109.591 |
| 11B | 0.014-6.034 | 2 | 92.753 | 10 | 93.363 | 40 | 88.732 |
| 27Al | 0.853-41.905 | 2 | 96.140 | 10 | 101.524 | 40 | 100.020 |
| 69Ga | 0.014-1.946 | 2 | 102.791 | 10 | 104.630 | 40 | 102.393 |
| 85Rb | 0.035-38.381 | 2 | 97.805 | 10 | 116.116 | 40 | 104.052 |
| 133Cs | 0.005-0.202 | 2 | 101.473 | 10 | 101.625 | 40 | 116.978 |
| 137Ba | 0.066-9.212 | 2 | 112.620 | 10 | 108.145 | 40 | 106.497 |

Table S3 Cox proportional hazard analysis for the association between each element and survival time of patients with ESCC

| Element | Variables | HR | 95.0% CI | | P |
| --- | --- | --- | --- | --- | --- |
|  |  |  | Lower | Upper |  |
| Ti | Gender |  |  |  | 0.016 |
|  | Female | 1 |  |  |  |
|  | Male | 1.605 | 1.094 | 2.355 |  |
|  | Age,year |  |  |  | 0.696 |
|  | <60 | 1 |  |  |  |
|  | ≥60 | 0.942 | 0.696 | 1.274 |  |
|  | Tumor location |  |  |  | 0.795 |
|  | Cervical esophagus-upper thoracic | 1 |  |  |  |
|  | Middle thoracic | 0.879 | 0.593 | 1.303 |  |
|  | Lower thoracic | 0.943 | 0.612 | 1.454 |  |
|  | Surgery |  |  |  | 0.002 |
|  | Yes | 1 |  |  |  |
|  | No | 1.823 | 1.254 | 2.649 |  |
|  | Clinical/Pathological Stage |  |  |  | <0.001 |
|  | I-II | 1 |  |  |  |
|  | III-IV | 2.232 | 1.587 | 3.138 |  |
|  | Ti |  |  |  | 0.534 |
|  | Tertile1 | 1 |  |  |  |
|  | Tertile2 | 1.104 | 0.768 | 1.586 |  |
|  | Tertile3 | 0.897 | 0.629 | 1.277 |  |
| V | Gender |  |  |  | 0.025 |
|  | Female | 1 |  |  |  |
|  | Male | 1.549 | 1.058 | 2.268 |  |
|  | Age,year |  |  |  | 0.715 |
|  | <60 | 1 |  |  |  |
|  | ≥60 | 0.945 | 0.698 | 1.28 |  |
|  | Tumor location |  |  |  | 0.816 |
|  | Cervical esophagus-upper thoracic | 1 |  |  |  |
|  | Middle thoracic | 0.885 | 0.594 | 1.319 |  |
|  | Lower thoracic | 0.947 | 0.611 | 1.466 |  |
|  | Surgery |  |  |  | 0.002 |
|  | Yes | 1 |  |  |  |
|  | No | 1.777 | 1.224 | 2.58 |  |
|  | Clinical/Pathological Stage |  |  |  | <0.001 |
|  | I-II | 1 |  |  |  |
|  | III-IV | 2.232 | 1.585 | 3.142 |  |
|  | V |  |  |  | 0.985 |
|  | Tertile1 | 1 |  |  |  |
|  | Tertile2 | 1.022 | 0.699 | 1.495 |  |
|  | Tertile3 | 0.993 | 0.676 | 1.458 |  |
| Cr | Gender |  |  |  | 0.026 |
|  | Female | 1 |  |  |  |
|  | Male | 1.537 | 1.052 | 2.245 |  |
|  | Age,year |  |  |  | 0.682 |
|  | <60 | 1 |  |  |  |
|  | ≥60 | 0.939 | 0.693 | 1.271 |  |
|  | Tumor location |  |  |  | 0.72 |
|  | Cervical esophagus-upper thoracic | 1 |  |  |  |
|  | Middle thoracic | 0.851 | 0.569 | 1.272 |  |
|  | Lower thoracic | 0.915 | 0.591 | 1.418 |  |
|  | Surgery |  |  |  | 0.003 |
|  | Yes | 1 |  |  |  |
|  | No | 1.761 | 1.212 | 2.561 |  |
|  | Clinical/Pathological Stage |  |  |  | <0.001 |
|  | I-II | 1 |  |  |  |
|  | III-IV | 2.277 | 1.615 | 3.21 |  |
|  | Cr |  |  |  | 0.582 |
|  | Tertile1 | 1 |  |  |  |
|  | Tertile2 | 0.933 | 0.648 | 1.344 |  |
|  | Tertile3 | 0.826 | 0.572 | 1.191 |  |
| Mn | Gender |  |  |  | 0.024 |
|  | Female | 1 |  |  |  |
|  | Male | 1.544 | 1.058 | 2.253 |  |
|  | Age,year |  |  |  | 0.741 |
|  | <60 | 1 |  |  |  |
|  | ≥60 | 0.95 | 0.703 | 1.285 |  |
|  | Tumor location |  |  |  | 0.705 |
|  | Cervical esophagus-upper thoracic | 1 |  |  |  |
|  | Middle thoracic | 0.85 | 0.573 | 1.26 |  |
|  | Lower thoracic | 0.918 | 0.597 | 1.411 |  |
|  | Surgery |  |  |  | 0.006 |
|  | Yes | 1 |  |  |  |
|  | No | 1.695 | 1.168 | 2.46 |  |
|  | Clinical/Pathological Stage |  |  |  | <0.001 |
|  | I-II | 1 |  |  |  |
|  | III-IV | 2.272 | 1.614 | 3.199 |  |
|  | Mn |  |  |  | 0.076 |
|  | Tertile1 | 1 |  |  |  |
|  | Tertile2 | 1.49 | 1.032 | 2.15 |  |
|  | Tertile3 | 1.127 | 0.768 | 1.655 |  |
| Co | Gender |  |  |  | 0.018 |
|  | Female | 1 |  |  |  |
|  | Male | 1.575 | 1.081 | 2.294 |  |
|  | Age,year |  |  |  | 0.728 |
|  | <60 | 1 |  |  |  |
|  | ≥60 | 0.948 | 0.701 | 1.281 |  |
|  | Tumor location |  |  |  | 0.842 |
|  | Cervical esophagus-upper thoracic | 1 |  |  |  |
|  | Middle thoracic | 0.901 | 0.607 | 1.338 |  |
|  | Lower thoracic | 0.969 | 0.628 | 1.494 |  |
|  | Surgery |  |  |  | 0.001 |
|  | Yes | 1 |  |  |  |
|  | No | 1.826 | 1.26 | 2.647 |  |
|  | Clinical/Pathological Stage |  |  |  | <0.001 |
|  | I-II | 1 |  |  |  |
|  | III-IV | 2.289 | 1.627 | 3.22 |  |
|  | Co |  |  |  | 0.039 |
|  | Tertile1 | 1 |  |  |  |
|  | Tertile2 | 1.216 | 0.835 | 1.771 |  |
|  | Tertile3 | 1.591 | 1.105 | 2.291 |  |
| Ni | Gender |  |  |  | 0.01 |
|  | Female | 1 |  |  |  |
|  | Male | 1.647 | 1.125 | 2.411 |  |
|  | Age,year |  |  |  | 0.789 |
|  | <60 | 1 |  |  |  |
|  | ≥60 | 0.96 | 0.71 | 1.298 |  |
|  | Tumor location |  |  |  | 0.743 |
|  | Cervical esophagus-upper thoracic | 1 |  |  |  |
|  | Middle thoracic | 0.869 | 0.588 | 1.284 |  |
|  | Lower thoracic | 0.949 | 0.616 | 1.464 |  |
|  | Surgery |  |  |  | 0.002 |
|  | Yes | 1 |  |  |  |
|  | No | 1.819 | 1.253 | 2.641 |  |
|  | Clinical/Pathological Stage |  |  |  | <0.001 |
|  | I-II | 1 |  |  |  |
|  | III-IV | 2.279 | 1.614 | 3.22 |  |
|  | Ni |  |  |  | 0.035 |
|  | Tertile1 | 1 |  |  |  |
|  | Tertile2 | 0.903 | 0.618 | 1.319 |  |
|  | Tertile3 | 1.404 | 0.977 | 2.017 |  |
| Cu | Gender |  |  |  | 0.02 |
|  | Female | 1 |  |  |  |
|  | Male | 1.568 | 1.075 | 2.288 |  |
|  | Age,year |  |  |  | 0.805 |
|  | <60 | 1 |  |  |  |
|  | ≥60 | 0.962 | 0.71 | 1.304 |  |
|  | Tumor location |  |  |  | 0.84 |
|  | Cervical esophagus-upper thoracic | 1 |  |  |  |
|  | Middle thoracic | 0.893 | 0.602 | 1.324 |  |
|  | Lower thoracic | 0.948 | 0.615 | 1.46 |  |
|  | Surgery |  |  |  | 0.005 |
|  | Yes | 1 |  |  |  |
|  | No | 1.717 | 1.179 | 2.5 |  |
|  | Clinical/Pathological Stage |  |  |  | <0.001 |
|  | I-II | 1 |  |  |  |
|  | III-IV | 2.2 | 1.559 | 3.104 |  |
|  | Cu |  |  |  | 0.533 |
|  | Tertile1 | 1 |  |  |  |
|  | Tertile2 | 1.082 | 0.746 | 1.57 |  |
|  | Tertile3 | 1.232 | 0.848 | 1.789 |  |
| Sr | Gender |  |  |  | 0.02 |
|  | Female | 1 |  |  |  |
|  | Male | 1.571 | 1.074 | 2.297 |  |
|  | Age,year |  |  |  | 0.59 |
|  | <60 | 1 |  |  |  |
|  | ≥60 | 0.919 | 0.676 | 1.249 |  |
|  | Tumor location |  |  |  | 0.817 |
|  | Cervical esophagus-upper thoracic | 1 |  |  |  |
|  | Middle thoracic | 0.888 | 0.599 | 1.318 |  |
|  | Lower thoracic | 0.953 | 0.618 | 1.47 |  |
|  | Surgery |  |  |  | 0.004 |
|  | Yes | 1 |  |  |  |
|  | No | 1.767 | 1.201 | 2.599 |  |
|  | Clinical/Pathological Stage |  |  |  | <0.001 |
|  | I-II | 1 |  |  |  |
|  | III-IV | 2.242 | 1.593 | 3.155 |  |
|  | Sr |  |  |  | 0.666 |
|  | Tertile1 | 1 |  |  |  |
|  | Tertile2 | 1.055 | 0.728 | 1.527 |  |
|  | Tertile3 | 0.895 | 0.614 | 1.305 |  |
| Cd | Gender |  |  |  | 0.027 |
|  | Female | 1 |  |  |  |
|  | Male | 1.53 | 1.049 | 2.232 |  |
|  | Age,year |  |  |  | 0.967 |
|  | <60 | 1 |  |  |  |
|  | ≥60 | 0.994 | 0.733 | 1.346 |  |
|  | Tumor location |  |  |  | 0.678 |
|  | Cervical esophagus-upper thoracic | 1 |  |  |  |
|  | Middle thoracic | 0.863 | 0.583 | 1.277 |  |
|  | Lower thoracic | 0.972 | 0.63 | 1.5 |  |
|  | Surgery |  |  |  | 0.001 |
|  | Yes | 1 |  |  |  |
|  | No | 1.913 | 1.317 | 2.779 |  |
|  | Clinical/Pathological Stage |  |  |  | <0.001 |
|  | I-II | 1 |  |  |  |
|  | III-IV | 2.23 | 1.577 | 3.152 |  |
|  | Cd |  |  |  | 0.007 |
|  | Tertile1 | 1 |  |  |  |
|  | Tertile2 | 1.372 | 0.939 | 2.004 |  |
|  | Tertile3 | 1.851 | 1.258 | 2.723 |  |
| Tl | Gender |  |  |  | 0.015 |
|  | Female | 1 |  |  |  |
|  | Male | 1.61 | 1.097 | 2.364 |  |
|  | Age,year |  |  |  | 0.668 |
|  | <60 | 1 |  |  |  |
|  | ≥60 | 0.936 | 0.691 | 1.267 |  |
|  | Tumor location |  |  |  | 0.716 |
|  | Cervical esophagus-upper thoracic | 1 |  |  |  |
|  | Middle thoracic | 0.854 | 0.575 | 1.269 |  |
|  | Lower thoracic | 0.926 | 0.601 | 1.427 |  |
|  | Surgery |  |  |  | 0.002 |
|  | Yes | 1 |  |  |  |
|  | No | 1.783 | 1.226 | 2.594 |  |
|  | Clinical/Pathological Stage |  |  |  | <0.001 |
|  | I-II | 1 |  |  |  |
|  | III-IV | 2.197 | 1.558 | 3.097 |  |
|  | Tl |  |  |  | 0.479 |
|  | Tertile1 | 1 |  |  |  |
|  | Tertile2 | 0.842 | 0.586 | 1.209 |  |
|  | Tertile3 | 0.804 | 0.555 | 1.165 |  |
| Pb | Gender |  |  |  | 0.021 |
|  | Female | 1 |  |  |  |
|  | Male | 1.568 | 1.072 | 2.293 |  |
|  | Age,year |  |  |  | 0.596 |
|  | <60 | 1 |  |  |  |
|  | ≥60 | 0.922 | 0.682 | 1.246 |  |
|  | Tumor location |  |  |  | 0.737 |
|  | Cervical esophagus-upper thoracic | 1 |  |  |  |
|  | Middle thoracic | 0.859 | 0.578 | 1.276 |  |
|  | Lower thoracic | 0.925 | 0.598 | 1.428 |  |
|  | Surgery |  |  |  | 0.002 |
|  | Yes | 1 |  |  |  |
|  | No | 1.816 | 1.249 | 2.639 |  |
|  | Clinical/Pathological Stage |  |  |  | <0.001 |
|  | I-II | 1 |  |  |  |
|  | III-IV | 2.265 | 1.612 | 3.183 |  |
|  | Pb |  |  |  | 0.09 |
|  | Tertile1 | 1 |  |  |  |
|  | Tertile2 | 1.05 | 0.73 | 1.51 |  |
|  | Tertile3 | 1.447 | 1.005 | 2.085 |  |
| B | Gender |  |  |  | 0.017 |
|  | Female | 1 |  |  |  |
|  | Male | 1.589 | 1.087 | 2.323 |  |
|  | Age,year |  |  |  | 0.746 |
|  | <60 | 1 |  |  |  |
|  | ≥60 | 0.951 | 0.703 | 1.287 |  |
|  | Tumor location |  |  |  | 0.896 |
|  | Cervical esophagus-upper thoracic | 1 |  |  |  |
|  | Middle thoracic | 0.923 | 0.618 | 1.379 |  |
|  | Lower thoracic | 0.982 | 0.632 | 1.526 |  |
|  | Surgery |  |  |  | 0.002 |
|  | Yes | 1 |  |  |  |
|  | No | 1.821 | 1.253 | 2.646 |  |
|  | Clinical/Pathological Stage |  |  |  | <0.001 |
|  | I-II | 1 |  |  |  |
|  | III-IV | 2.209 | 1.569 | 3.11 |  |
|  | B |  |  |  | 0.329 |
|  | Tertile1 | 1 |  |  |  |
|  | Tertile2 | 0.802 | 0.563 | 1.141 |  |
|  | Tertile3 | 0.787 | 0.548 | 1.13 |  |
| Al | Gender |  |  |  | 0.025 |
|  | Female | 1 |  |  |  |
|  | Male | 1.547 | 1.056 | 2.266 |  |
|  | Age,year |  |  |  | 0.656 |
|  | <60 | 1 |  |  |  |
|  | ≥60 | 0.934 | 0.69 | 1.263 |  |
|  | Tumor location |  |  |  | 0.715 |
|  | Cervical esophagus-upper thoracic | 1 |  |  |  |
|  | Middle thoracic | 0.851 | 0.57 | 1.27 |  |
|  | Lower thoracic | 0.92 | 0.595 | 1.421 |  |
|  | Surgery |  |  |  | 0.004 |
|  | Yes | 1 |  |  |  |
|  | No | 1.743 | 1.199 | 2.532 |  |
|  | Clinical/Pathological Stage |  |  |  | <0.001 |
|  | I-II | 1 |  |  |  |
|  | III-IV | 2.245 | 1.595 | 3.161 |  |
|  | Al |  |  |  | 0.62 |
|  | Tertile1 | 1 |  |  |  |
|  | Tertile2 | 0.89 | 0.62 | 1.277 |  |
|  | Tertile3 | 1.074 | 0.753 | 1.532 |  |
| Ga | Gender |  |  |  | 0.015 |
|  | Female | 1 |  |  |  |
|  | Male | 1.6 | 1.095 | 2.337 |  |
|  | Age,year |  |  |  | 0.81 |
|  | <60 | 1 |  |  |  |
|  | ≥60 | 0.964 | 0.712 | 1.304 |  |
|  | Tumor location |  |  |  | 0.904 |
|  | Cervical esophagus-upper thoracic | 1 |  |  |  |
|  | Middle thoracic | 0.915 | 0.617 | 1.356 |  |
|  | Lower thoracic | 0.926 | 0.603 | 1.423 |  |
|  | Surgery |  |  |  | 0.001 |
|  | Yes | 1 |  |  |  |
|  | No | 1.835 | 1.262 | 2.668 |  |
|  | Clinical/Pathological Stage |  |  |  | <0.001 |
|  | I-II | 1 |  |  |  |
|  | III-IV | 2.291 | 1.626 | 3.228 |  |
|  | Ga |  |  |  | 0.042 |
|  | Tertile1 | 1 |  |  |  |
|  | Tertile2 | 0.719 | 0.51 | 1.014 |  |
|  | Tertile3 | 0.64 | 0.438 | 0.934 |  |
| Rb | Gender |  |  |  | 0.011 |
|  | Female | 1 |  |  |  |
|  | Male | 1.64 | 1.12 | 2.402 |  |
|  | Age,year |  |  |  | 0.781 |
|  | <60 | 1 |  |  |  |
|  | ≥60 | 0.958 | 0.708 | 1.296 |  |
|  | Tumor location |  |  |  | 0.722 |
|  | Cervical esophagus-upper thoracic | 1 |  |  |  |
|  | Middle thoracic | 0.859 | 0.581 | 1.27 |  |
|  | Lower thoracic | 0.936 | 0.609 | 1.437 |  |
|  | Surgery |  |  |  | 0.001 |
|  | Yes | 1 |  |  |  |
|  | No | 1.832 | 1.268 | 2.646 |  |
|  | Clinical/Pathological Stage |  |  |  | <0.001 |
|  | I-II | 1 |  |  |  |
|  | III-IV | 2.279 | 1.619 | 3.209 |  |
|  | Rb |  |  |  | 0.019 |
|  | Tertile1 | 1 |  |  |  |
|  | Tertile2 | 0.591 | 0.408 | 0.857 |  |
|  | Tertile3 | 0.83 | 0.584 | 1.181 |  |
| Cs | Gender |  |  |  | 0.024 |
|  | Female | 1 |  |  |  |
|  | Male | 1.55 | 1.06 | 2.268 |  |
|  | Age,year |  |  |  | 0.671 |
|  | <60 | 1 |  |  |  |
|  | ≥60 | 0.936 | 0.69 | 1.27 |  |
|  | Tumor location |  |  |  | 0.782 |
|  | Cervical esophagus-upper thoracic | 1 |  |  |  |
|  | Middle thoracic | 0.878 | 0.592 | 1.302 |  |
|  | Lower thoracic | 0.951 | 0.616 | 1.466 |  |
|  | Surgery |  |  |  | 0.002 |
|  | Yes | 1 |  |  |  |
|  | No | 1.808 | 1.239 | 2.637 |  |
|  | Clinical/Pathological Stage |  |  |  | <0.001 |
|  | I-II | 1 |  |  |  |
|  | III-IV | 2.264 | 1.604 | 3.196 |  |
|  | Cs |  |  |  | 0.79 |
|  | Tertile1 | 1 |  |  |  |
|  | Tertile2 | 1.067 | 0.732 | 1.556 |  |
|  | Tertile3 | 0.94 | 0.657 | 1.345 |  |
| Ba | Gender |  |  |  | 0.013 |
|  | Female | 1 |  |  |  |
|  | Male | 1.616 | 1.106 | 2.361 |  |
|  | Age,year |  |  |  | 0.867 |
|  | <60 | 1 |  |  |  |
|  | ≥60 | 0.974 | 0.719 | 1.32 |  |
|  | Tumor location |  |  |  | 0.887 |
|  | Cervical esophagus-upper thoracic | 1 |  |  |  |
|  | Middle thoracic | 0.91 | 0.614 | 1.349 |  |
|  | Lower thoracic | 0.954 | 0.621 | 1.468 |  |
|  | Surgery |  |  |  | 0.001 |
|  | Yes | 1 |  |  |  |
|  | No | 1.859 | 1.278 | 2.704 |  |
|  | Clinical/Pathological Stage |  |  |  | <0.001 |
|  | I-II | 1 |  |  |  |
|  | III-IV | 2.319 | 1.646 | 3.267 |  |
|  | Ba |  |  |  | 0.034 |
|  | Tertile1 | 1 |  |  |  |
|  | Tertile2 | 0.679 | 0.48 | 0.961 |  |
|  | Tertile3 | 0.659 | 0.455 | 0.956 |  |

Table S4 Total variance explained

| Factors | Extraction Sums of Squared Loadings | | |  | Rotation Sums of Squared Loadings | | |
| --- | --- | --- | --- | --- | --- | --- | --- |
|  | Total | Variance (%) | Cumulative (%) |  | Total | Variance (%) | Cumulative (%) |
| 1 | 3.421 | 20.124 | 20.124 |  | 2.652 | 15.597 | 15.597 |
| 2 | 2.799 | 16.462 | 36.586 |  | 2.455 | 14.441 | 30.038 |
| 3 | 2.114 | 12.434 | 49.02 |  | 2.115 | 12.439 | 42.477 |
| 4 | 1.404 | 8.258 | 57.278 |  | 1.842 | 10.835 | 53.312 |
| 5 | 1.234 | 7.258 | 64.536 |  | 1.488 | 8.755 | 62.067 |
| 6 | 1.011 | 5.946 | 70.482 |  | 1.431 | 8.415 | 70.482 |

Table S5 Cox proportional hazard analysis for the association between extracting common factors and survival time of patients with ESCC

| Variables | HR | 95.0% CI | | P |
| --- | --- | --- | --- | --- |
|  |  | Lower | Upper |  |
| Gender |  |  |  |  |
| Female | 1 |  |  |  |
| Male | 1.664 | 1.133 | 2.442 | 0.009 |
| Age,year |  |  |  |  |
| <60 | 1 |  |  |  |
| ≥60 | 0.899 | 0.661 | 1.224 | 0.499 |
| Tumor location |  |  |  |  |
| Cervical esophagus-upper thoracic | 1 |  |  |  |
| Middle thoracic | 0.912 | 0.608 | 1.369 | 0.657 |
| Lower thoracic | 0.973 | 0.626 | 1.511 | 0.902 |
| Surgery |  |  |  |  |
| Yes | 1 |  |  |  |
| No | 2.235 | 1.505 | 3.319 | <0.001 |
| Clinical/Pathological Stage |  |  |  |  |
| I-II | 1 |  |  |  |
| III-IV | 2.337 | 1.65 | 3.311 | <0.001 |
| Factor1 |  |  |  |  |
| Tertile 1 | 1 |  |  |  |
| Tertile 2 | 0.552 | 0.383 | 0.794 | 0.001 |
| Tertile 3 | 0.624 | 0.428 | 0.909 | 0.014 |
| Factor2 |  |  |  |  |
| Tertile 1 | 1 |  |  |  |
| Tertile 2 | 1.324 | 0.916 | 1.914 | 0.136 |
| Tertile 3 | 1.447 | 0.987 | 2.123 | 0.058 |
| Factor3 |  |  |  |  |
| Tertile 1 | 1 |  |  |  |
| Tertile 2 | 0.957 | 0.652 | 1.406 | 0.824 |
| Tertile 3 | 0.998 | 0.682 | 1.46 | 0.99 |
| Factor4 |  |  |  |  |
| Tertile 1 | 1 |  |  |  |
| Tertile 2 | 0.896 | 0.618 | 1.298 | 0.561 |
| Tertile 3 | 1.063 | 0.725 | 1.558 | 0.755 |
| Factor5 |  |  |  |  |
| Tertile 1 | 1 |  |  |  |
| Tertile 2 | 0.674 | 0.446 | 1.017 | 0.06 |
| Tertile 3 | 0.971 | 0.663 | 1.423 | 0.882 |
| Factor6 |  |  |  |  |
| Tertile 1 | 1 |  |  |  |
| Tertile 2 | 0.836 | 0.576 | 1.213 | 0.345 |
| Tertile 3 | 1.014 | 0.687 | 1.497 | 0.943 |

Table S6 PIP value of each element in factor 1

|  | 1-year | 3-year | 5-year |
| --- | --- | --- | --- |
| Ga | 0.6092 | 0.7842 | 0.8144 |
| Ba | 0.4718 | 0.8000 | 0.8460 |
| B | 0.3038 | 0.5876 | 0.4880 |

Table S7 PIP value of each element in factor 2

|  | 1-year | 3-year | 5-year |
| --- | --- | --- | --- |
| Co | 0.2208 | 0.1836 | 0.3840 |
| Ti | 0.3100 | 0.2166 | 0.3664 |
| Cd | 0.9354 | 0.2336 | 0.8768 |
| Pb | 0.3516 | 0.2530 | 0.3632 |
