# Supplementary figures and images for: Effect of trace element mixtures on the outcome of patients with esophageal squamous cell carcinoma: a prospective cohort study in Fujian, China
Source: BMC Cancer. 2024 Jan 2;24:24. doi: 10.1186/s12885-023-11763-9 (PMC10762846; doi:10.1186/s12885-023-11763-9)

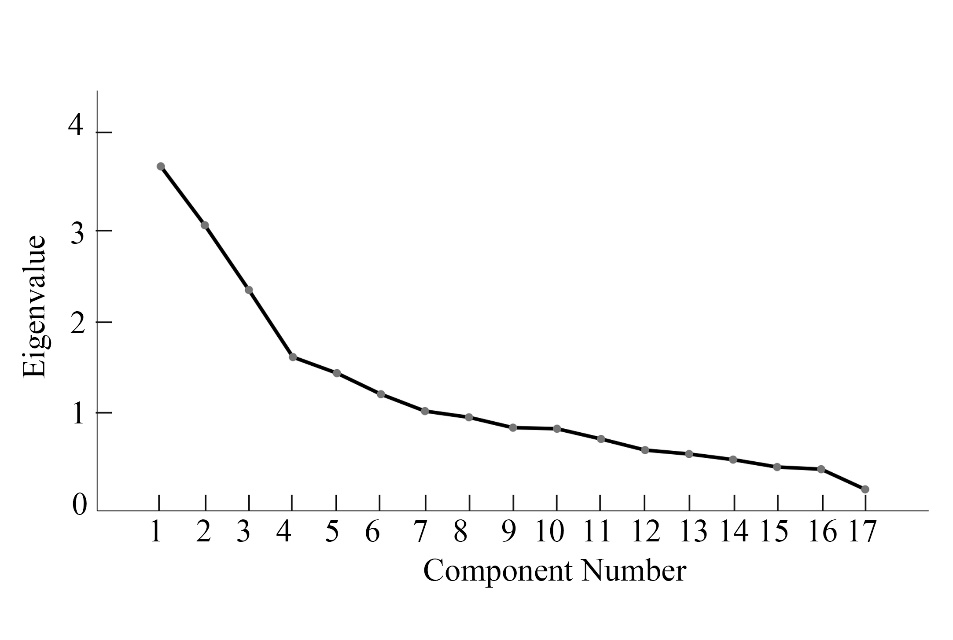


FigS1 Scree plot

Supplement: Supplementary file 2 — Additional file 2: Fig. S1. Scree plot. [file 12885_2023_11763_MOESM2_ESM.docx]
